# Transcriptomic Profiling Provides Molecular Insights Into Hydrogen Peroxide-Enhanced *Arabidopsis* Growth and Its Salt Tolerance

**DOI:** 10.3389/fpls.2022.866063

**Published:** 2022-04-06

**Authors:** Qikun Zhang, Xiuru Dai, Huanpeng Wang, Fanhua Wang, Dongxue Tang, Chunyun Jiang, Xiaoyan Zhang, Wenjing Guo, Yuanyuan Lei, Changle Ma, Hui Zhang, Pinghua Li, Yanxiu Zhao, Zenglan Wang

**Affiliations:** ^1^Shandong Provincial Key Laboratory of Plant Stress, College of Life Sciences, Shandong Normal University, Jinan, China; ^2^State Key Laboratory of Crop Biology, College of Agronomic Sciences, Shandong Agricultural University, Tai’an, China; ^3^Linyi Center for Disease Control and Prevention, Linyi, China

**Keywords:** hydrogen peroxide, pretreatment, *Arabidopsis thaliana*, salt stress, transcriptome profiling

## Abstract

Salt stress is an important environmental factor limiting plant growth and crop production. Plant adaptation to salt stress can be improved by chemical pretreatment. This study aims to identify whether hydrogen peroxide (H_2_O_2_) pretreatment of seedlings affects the stress tolerance of *Arabidopsis thaliana* seedlings. The results show that pretreatment with H_2_O_2_ at appropriate concentrations enhances the salt tolerance ability of Arabidopsis seedlings, as revealed by lower Na^+^ levels, greater K^+^ levels, and improved K^+^/Na^+^ ratios in leaves. Furthermore, H_2_O_2_ pretreatment improves the membrane properties by reducing the relative membrane permeability (RMP) and malonaldehyde (MDA) content in addition to improving the activities of antioxidant enzymes, including superoxide dismutase, and glutathione peroxidase. Our transcription data show that exogenous H_2_O_2_ pretreatment leads to the induced expression of cell cycle, redox regulation, and cell wall organization-related genes in Arabidopsis, which may accelerate cell proliferation, enhance tolerance to osmotic stress, maintain the redox balance, and remodel the cell walls of plants in subsequent high-salt environments.

## Introduction

Salt stress seriously influences plant growth, development, and crop yield (Deinlein et al., 2014; Gong et al., 2020; Zhao et al., 2020). High salinity can cause hyperosmotic stress, ion toxicity, nutrient deficiency, and subsequent oxidative damage due to the overproduction of reactive oxygen species (ROS) in plants, ultimately leading to plant cell dysfunction, growth inhibition, leaf senescence, and even plant death (Munns and Tester, 2008; Van Zelm et al., 2020). In order to adapt to salt stress, plants have developed a series of sophisticated physiological mechanisms, such as the adjustment of membrane systems, reconstruction of ionic and osmotic homeostasis, modification of cell wall structure, and maintenance of redox balance (Cramer et al., 2011; Van Zelm et al., 2020). In addition to these physiological mechanisms, there exist measures in production practice to increase salt tolerance, such as gene engineering, chemical pretreatment, and abiotic stress acclimation (Shen et al., 2014; Tian et al., 2018). Among the various strategies, chemical pretreatment, especially hydrogen peroxide (H_2_O_2_) pretreatment, is a very simple, low-cost, and effective approach to enhance plant tolerance to environmental stresses (Ashraf and Foolad, 2005; Beckers et al., 2009; Wahid and Shabbir, 2015).

H_2_O_2_ is the most stable component of ROS and has generally been considered to be a toxic cellular metabolite (Anjum et al., 2015). On the other hand, it can function as a signaling molecule in both animal and plant cells, adjusting their tolerance to adverse environments (Cerny et al., 2018). Several previous studies have reported that H_2_O_2_ may play a dual role in plants (Neill et al., 2002). At high concentrations, H_2_O_2_ can cause lipid peroxidation, protein disfunction, and programmed cell death. By contrast, at low concentrations, H_2_O_2_ acts as a messenger molecule that may directly regulate the expression of numerous genes and trigger the responses of plants to abiotic stresses (Vandenabeele et al., 2003; Petrov and Van Breusegem, 2012). Hence, H_2_O_2_ signaling is of potential significance in improving crop tolerance to environmental stresses.

Several studies have shown that the pretreatment of plants with exogenous H_2_O_2_ can significantly increase abiotic stress tolerance. For example, pretreatment of H_2_O_2_ protected *Arabidopsis thaliana* leaves against excess light damage (Karpinski et al., 1999), induced the adaptation of rice seedlings to salt stress and high temperature (Uchida et al., 2002), improved the salt resistances of barley (Fedina et al., 2009), maize (Gondim et al., 2012) and sunflower (Silva et al., 2020), enhanced the chilling tolerance of the two *Zoysia* cultivars Manila grass (*Zoysia matrella*) and Mascarene grass (*Zoysia tenuifolia*) (Wang et al., 2010), induced salt stress acclimation in maize plants (De Azevedo Neto et al., 2005), and alleviates drought stress in soybean plants (Ishibashi et al., 2011). The pretreatment of wheat seeds with H_2_O_2_ also enhanced the subsequent drought (He et al., 2009) and salt (Wahid et al., 2007) resistances of the seedlings. Additionally, H_2_O_2_ pretreatment protected tobacco from oxidative stresses generated by high light intensities or the catalase inhibitor aminotriazole through induction of a set of antioxidant enzymes (Gechev et al., 2002). Therefore, the accumulation of H_2_O_2_ in specific tissues and at appropriate levels could enhance the activities of antioxidant enzymes and, therefore, aid plants in adaptation to different unfavorable environmental cues (Bowler and Fluhr, 2000).

Although H_2_O_2_ pretreatment is important for improving plant salt tolerance, little is known about the mechanism of salt tolerance improvement by H_2_O_2_ pretreatment during the growth and development of plants.

In this study, 4-week-old Arabidopsis leaves were sprayed with H_2_O_2_ before being subjected to NaCl stress. We found that H_2_O_2_ pretreatment resulted in improvements to some physiological and biochemical responses of the Arabidopsis seedling to salt stress. To obtain insights into the molecular mechanisms of H_2_O_2_-induced salt tolerance, we then performed transcriptome profiling of the Arabidopsis seedlings under H_2_O_2_ pretreatment followed by salt stress. This work aims to understand the mechanisms of salt stress acclimation in Arabidopsis induced by H_2_O_2_.

## Materials and Methods

### Plant Materials and Growth Conditions

*Arabidopsis thaliana* ecotype Columbia (Col-0) was used in this study. Arabidopsis seeds were sterilized with 75% (v/v) ethanol and then washed with sterile distilled water. The seeds were then sown on half-strength Murashige and Skoog (MS) medium. After stratification for 3 days in a 4°C refrigerator, the plates were transferred to growth chambers. Eight days after seed germination, Arabidopsis seedlings were transferred into 9 cm diameter pots containing soil, perlite, and vermiculite (2:1:1), with irrigation of half-strength Hoagland’s nutrient solution. The growth condition in the growth chambers was a 16 h light/8 h dark photoperiod with a day/night thermoperiod of 22°C/18°C, a relative humidity of 70%, and irradiance of 110 μmol m^–2^ s^–1^.

### H_2_O_2_ Foliar Spraying of Arabidopsis Seedling Followed by NaCl Stress

Four-week-old seedlings were randomly divided into four groups, and the seedlings in each group were subjected to treatment as follows: pretreatment–stressed (pretreated with H_2_O_2_ and salt-stressed, HN); non-pretreatment–stressed (pretreated with water and salt-stressed, WN); pretreatment–non-stressed (pretreated with H_2_O_2_ and not salt-stressed, HW); and non-pretreatment–non-stressed (i.e., control; pretreated with water and not salt-stressed, WW). For pretreatment–stressed and pretreatment–non-stressed plants, leaves were sprayed with 20 μM of H_2_O_2_ solution four times at 4-h intervals, while non-pretreatment–stressed and non-pretreatment–non-stressed plants were sprayed with water. Twenty-four hours after foliar spraying, Arabidopsis seedlings from pretreatment–stressed and non-pretreatment–stressed groups were subsequently watered with 150 mM NaCl every day, whereas pretreatment–non-stressed and control plants were treated with water. Twelve hours after treatment with 150 mM NaCl, Arabidopsis seedlings were collected for transcriptome profiling analysis. Four days after treatment with 150 mM NaCl, Arabidopsis seedlings were collected for the determination of various physiological parameters.

### Measurement of Dry and Fresh Weight of Seedlings

The fresh weight of the shoots from each treatment was determined immediately after harvesting, and samples were dried in an oven at 70°C for 24 h to obtain dry weights. Twenty individual plants were collected for each replicate and triplicates were analyzed in parallel.

### Determination of Relative Membrane Permeability

The relative membrane permeability (RMP) of the seedlings was determined following the method of Yang et al. (1996). Excised fresh leaves (0.5 g) were immediately put into test tubes containing 10 mL of deionized distilled water and briefly vortexed. The solution was used to measure initial electrical conductivity (EC0). The test tubes containing leaves in distilled water were kept at 4°C for 24 h and EC1 was determined. The test tubes were then placed in a boiling water bath for 10 min, cooled to room temperature, and the boiled leachate was filtered and measured for EC2. RMP was computed using the following formula: RMP (%) = [(EC1 − EC0)/(EC2 − EC0)] × 100.

### Measurement of Malonaldehyde

The level of lipid peroxidation in the leaf tissue was measured in terms of MDA (a product of lipid peroxidation) content, detected by the thiobarbituric acid reaction using the method of Dhindsa et al. (1981). Fresh leaf samples (0.4 g) were homogenized in 5 mL of 0.1% trichloroacetic acid, vortexed, and then 4 mL of 0.5% thiobarbituric acid was added. The mixture was heated to 95°C for 30 min and was quickly cooled in an ice bath. Afterward, the mixture was centrifuged at 3000 rpm for 10 min. The supernatant fraction was collected, and the absorbance of the supernatant at 532 and 600 nm was read. The value for the non-specific absorption at 600 nm was subtracted from that at 532 nm (Zhang and Kirkham, 1996). The concentration of MDA was calculated using the extinction coefficient of MDA (155 mM^–1^ cm^–1^) (Heath and Packer, 1968) and expressed as nmol MDA g^–1^ fresh weight. Each treatment was carried out in triplicate.

### Determination of Na^+^ and K^+^ Content

Fifteen dry plants were pooled together and ground into fine powder; 0.02 g of dry powder was ashed in a muffle furnace at 300°C for 2 h, then 550°C for 10 h. The ash was resolved into small amounts of concentrated nitric acid and adjusted to a final volume of 10 mL. The ion content of samples was determined with a flame photometer (2655-00 Digital Flame Analyzer, Cole-Parmer Instrument Company, Chicago, IL, United States). Three independent determinations were performed for each treatment (Song et al., 2005).

### Transcriptome Profiling Analysis

Twelve hours after treatment with 150 mM NaCl, Arabidopsis seedlings from the four experimental groups (WW, WN, HW, and HN) were collected to extract total RNA using Biozol reagent (Bio Flux, Beijing, China) according to the manufacturer’s instructions. The integrity and quality of the isolated RNA were monitored by agarose gel electrophoresis. RNA concentration was quantified by a Nanodrop ND-1000 spectrophotometer (Thermo Scientific, Massachusetts, United States). The qualified RNA samples were sent to the Annoroad Gene Technology Corporation (Beijing, China), and the libraries were sequenced on an Illumina platform and 150 bp paired-end reads were generated. RNA-Seq data of the four experimental samples were obtained from three biological replicates, respectively. RNA-Seq data were deposited into the NCBI’s Sequence Read Archive (the accession number is PRJNA612654).

### Gene Ontology and Kyoto Encyclopedia of Genes and Genomes Enrichment Analysis

Low-quality reads were trimmed using Trimmomatic (Bolger et al., 2014) (v 0.36) with the settings “LEADING:3 TRAILING:3 SLIDINGWINDOW:4:15 MINLEN:36”. Clean reads were mapped to the Arabidopsis TAIR10 release obtained from TAIR^1^ using TopHat (v 2.1.1) with settings “-N 1 –num-threads 6”. Count data were generated by Cufflinks (v 2.2.1) and FPKM (fragments per kilobase per million mapped reads) was used to estimate the expression levels of individual genes.

Differentially expressed genes (DEGs) were identified by DESeq2 Moderated estimation of fold change and dispersion (Love et al., 2014) using the Bioconductor software^2^, based on a comparison across all samples under control or different experimental conditions with false discovery rate (FDR) less than 0.05. The Goatools (v 0.8.9) python package was used for GO term enrichment (Klopfenstein et al., 2018) with the Arabidopsis association files downloaded from TAIR10. Kyoto Encyclopedia of Genes and Genomes (KEGG) pathway enrichment analysis of genes was performed using clusterProfiler package (v 4.2.2, Wu et al., 2021).

### Reverse Transcription and Quantitative Real-Time PCR Analysis

Transcriptome profiling results were validated and verified by quantitative real-time PCR experiments, in which 2 μg of total RNA was used for reverse transcription to obtain the cDNA using FastQuant RT Kit (with gDNase, TIANGEN, Beijing, China). The SuperReal PreMix Plus Kit (SYBR Green, TIANGEN) was used along with the cDNA for quantitative real-time PCR experiments using a real-time fluorescence quantitative PCR instrument (LightCycler^®^ 96, Roche, Basel, Switzerland). All reactions were assayed using three replicates. *Actin2* was used as an endogenous control. The relative expression levels are presented as values relative to that of the corresponding control sample at the indicated time after normalization to *actin* transcript levels. Primer sequences are shown in the Supplementary Table 1.

### Measurement of Enzyme Activity

#### Extract Preparation

Frozen leaves (0.2 g) were crushed into a fine powder with a mortar and pestle in liquid N2. Soluble proteins were extracted by homogenizing the powder in 1 mL of 100 mM potassium phosphate buffer (pH 7.5) containing 2 mM EDTA, 1% (w/v) PVP-40, 10 mM DTT, and 1 mM PMSF. The homogenate was centrifuged at 12,000 rpm for 15 min and the supernatant fraction was used as a crude extract for enzyme activity. All operations were carried out at 4°C. The protein concentration was determined using the Bradford method (Bradford, 1976).

### Enzyme Activity Assays

#### Superoxide Dismutase

Total superoxide dismutase (SOD) activity was determined by measuring its ability to inhibit the photochemical reduction of nitro blue tetrazolium chloride (NBT), as described by Giannopolitis and Ries (1977). The reaction mixture (3 mL) contained 50 mM phosphate buffer (pH 7.8), 0.1 mM EDTA, 13 mM methionine, 75 μM NBT, 2 μM riboflavin, 0.05 M sodium carbonate (pH 10.2), and 100 μL enzyme extract. Riboflavin was added last and the tubes were shaken under fluorescent lamps at 110 μmol m^–2^ s^–1^. This reaction was allowed to proceed for 15 min, after which the lights were switched off and the tubes were covered with a black cloth. The absorbance of the reaction mixture was read at 560 nm. One unit of SOD activity (U) was defined as the amount of enzyme required to cause 50% inhibition of the NBT photoreduction rate. Results are expressed as units mg^–1^ protein per minute.

#### Glutathione Peroxidase

Total glutathione peroxidase (GPX) activity was determined as described by Drotar et al. (1985), with a reaction mixture (4 mL) containing 50 mM phosphate buffer (pH 7.0), 2.0 mM EDTA, 2.0 mM GSH, 0.1 mM NADPH, 2.5 units of glutathione reductase, and 100 μL enzyme extract; 0.09 mM H_2_O_2_ was added last to mark the beginning of the reaction. The reaction rate was measured by following the loss of NADPH spectrophotometrically at 340 nm. One unit of GPX activity was defined as the amount of enzyme that would cause the oxidation of 1.0 nmol of NADPH to NADP^+^ per minute at 25°C.

### Statistical Analysis

All the above experiments involved three biological replicates, and each experiment (except RNA-Seq) was carried out twice at different times. All data are expressed as means ± standard deviation and the significance of differences between datasets was evaluated by one-way ANOVA following SPSS. *P*-values of <0.05 were considered to be significantly different.

## Results

### Effect of H_2_O_2_ Foliar Spraying on Physiological Indices of Arabidopsis Seedlings

In order to evaluate the effects of H_2_O_2_ pretreatment on Arabidopsis growth under salinity, we sprayed the leaves of Arabidopsis seedling with 20 μM H_2_O_2_ and subsequently exposed them to 150 mM NaCl. We then determined some physiological indices as shown below.

#### Dry and Fresh Weight

**FIGURE 1:**
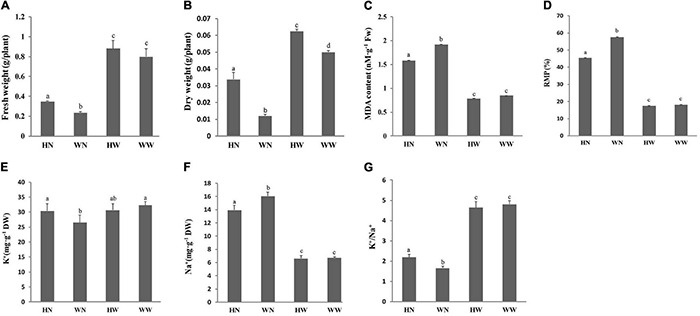
Physiological response of Arabidopsis seedlings to salt stress after H_2_O_2_ pretreatment. The influence of H_2_O_2_ pretreatment on the aerial fresh weight **(A)** and dry weight **(B)** of Arabidopsis seedlings under different treatments; the impact of H_2_O_2_ pretreatment on MDA content **(C)** and RMP **(D)** of Arabidopsis leaves under different treatments; and the impact of H_2_O_2_ pretreatment on K^+^ **(E)** and Na^+^ **(F)** content and K^+^/Na^+^ **(G)** in leaves of Arabidopsis seedlings under different treatments. Data are represented as means ± SD. Three biological replicates per experiment. Means for each treatment that do not share a common letter are significantly different at *P* < 0.05, estimated with one-way ANOVA following SPSS. WW, pretreated with water and not salt-stressed; WN, pretreated with water and salt-stressed; HW, pretreated with H_2_O_2_ and not salt-stressed; HN, pretreated with H_2_O_2_ and salt-stressed.

Data of shoot fresh weight and dry mass are shown in Figures 1A,B, respectively. Compared with controls, the pretreatment of seedlings with H_2_O_2_ significantly increased the aerial dry and fresh weight, regardless of the stress conditions. Although the salt-stressed plants had reduced shoot dry mass and fresh weight compared to unstressed plants, the growth inhibition caused by the salt stress decreased when the seedlings were sprayed with H_2_O_2_. Compared with WN plants, the HN plants increased shoot fresh weight and dry weight by 48.4 and 181.25%, respectively.

#### Effect of H_2_O_2_ Pretreatment on Malonaldehyde Content and the Relative Membrane Permeability of the Leaves

There were multiple significant differences in MDA content between the HN and WN groups under salt stress conditions. Compared to the WW group (control), the HW group had a slightly lower MDA content, however, the difference was not significant (Figure 1C). These results indicate that H_2_O_2_ pretreatment can reduce membrane lipid peroxidation of plant cells and, therefore, maintain the stability of the membrane.

Relative membrane permeability was greatly increased due to salinity, while the pretreatment of seedlings with 20 μM H_2_O_2_ reduced the RMP of corresponding seedlings under salt stress. Thus, the RMP of the WN group was higher than that of the HN group (Figure 1D).

#### Impact of H_2_O_2_ Pretreatment on Na^+^, K^+^ Content and K^+^/Na^+^ Ratio of Arabidopsis Shoots

Compared with the WN group, the seedlings in the HN group contained higher K^+^ (Figure 1E) and lower Na^+^ (Figure 1F) levels under the same salinity, which indicates that H_2_O_2_ pretreatment improved K^+^ uptake and K^+^/Na^+^ (Figure 1G) of Arabidopsis under salt stress conditions, thereby reducing the harm caused by Na^+^ to the plant.

### Transcriptome Profiling Analysis

In order to analyze the molecular mechanisms of salt tolerance improvement induced by H_2_O_2_ pretreatment, we collected the leaves of 4-week-old Arabidopsis seedlings treated with HN, WN, HW, and WW (with WW being the control) and the total RNA was extracted for genome-wide transcriptome analysis. RNA-Seq data were analyzed from a total of twelve samples comprising three biological replicates for each treatment.

**FIGURE 2:**
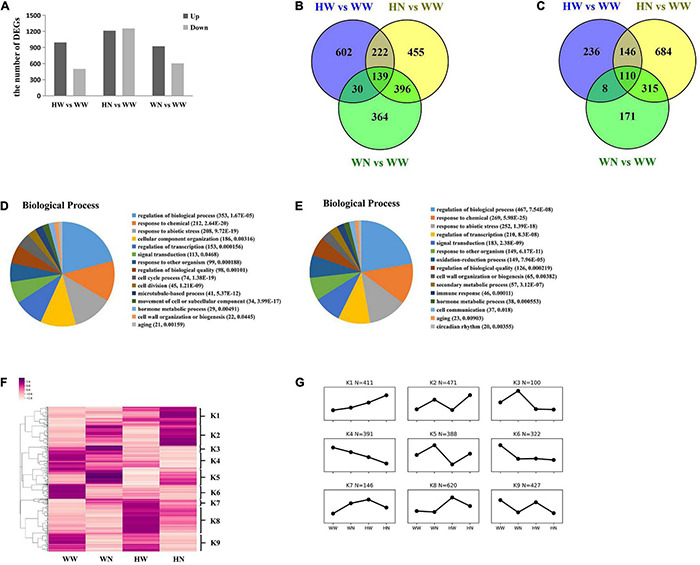
Transcriptome analysis of Arabidopsis leaves under WW, WN, HW, and HN treatments. **(A)** In HW vs. WW, 993 DEGs were up-regulated, whereas 500 DEGs were down-regulated. In HN vs. WW, 1212 DEGs were up-regulated, whereas 1255 DEGs were down-regulated. In WN vs. WW, 922 DEGs were up-regulated, whereas 604 DEGs were down-regulated. **(B)** Venn diagram of DEGs up-regulated in WN vs. WW, HW vs. WW, and HN vs. WW. There were 602 unique DEGs in HW vs. WW, 455 unique DEGs in HN vs. WW, and 364 unique DEGs in WN vs. WW. There were 361 DEGs common to both HW and HN, 169 DEGs common to both HW and WN, and 535 DEGs common to both HN and WN. **(C)** Venn diagram of DEGs down-regulated in WN vs. WW, HW vs. WW, and HN vs. WW. **(D)** Biological process in HW vs. WW; **(E)** biological process in HN vs. WW. DEGs were identified by DESeq2 using Bioconductor (http://www.bioconductor.org/) based on a comparison across all samples under control or HW and control or HN conditions with FDR less than 0.05. The Goatools (v0.8.9) python package was used for GO terms enrichment with the Arabidopsis association files downloaded from TAIR10. **(F)** 1493 DEGs in HW vs. WW and 1766 DEGs in HN vs. WW clustered by heat-mapping under WW, WN, HW, and HN treatments (*p* ≤ 0.05). **(G)** Nine groups of genes were identified based on the heatmap dendrogram. Averaged values for each condition in every group were used to generate the line chart, which represents the comprehensive expression patterns of each group. WW, pretreated with water and not salt-stressed; WN, pretreated with water and salt-stressed; HW, pretreated with H_2_O_2_ and not salt-stressed; HN, pretreated with H_2_O_2_ and salt-stressed.

In total, 19,391 genes were detected in the leaves of Arabidopsis. We further obtained 1493 DEGs in HW, compared to WW, with at least twofold change of gene expression at *P*-value < 0.05. Among these, 993 genes were up-regulated and 500 genes were down-regulated (Figure 2A and Supplementary Table 2). Similarly, of the 2467 DEGs specifically responding to HN treatment in comparison to WW, 1212 genes were up-regulated whereas 1255 genes were down-regulated (Figure 2A and Supplementary Table 3). Among the 1533 DEGs in WN compared to WW, 922 genes were up-regulated while 604 genes were down-regulated (Figure 2A and Supplementary Table 4). We performed a preliminary analysis of up-regulated (Figure 2B) and down-regulated (Figure 2C) genes through Venn diagrams of HN vs. WW, HW vs. WW, and WN vs. WW. We found 602 unique DEGs in HW vs. WW (Supplementary Table 5), 455 unique DEGs in HN vs. WW (Supplementary Table 6), and 364 unique DEGs in WN vs. WW (Supplementary Table 7). There were an additional 361 DEGs that were common to both HW vs. WW and HN vs. WW (Supplementary Table 8), 169 DEGs common to both HW vs. WW and WN vs. WW (Supplementary Table 9), and 535 DEGs common to both HN vs. WW and WN vs. WW (Supplementary Table 10). Relevant data on down-regulated genes are also presented (Figure 2C and Supplementary Tables S11–16).

In order to rule out that some DEGs may have only been influenced by salt stress in HN vs. WW, we removed the DEGs from HN vs. WW that did not have a large difference in abundance with WN vs. WW; that is, those genes whose ratio of log_2_FC (HN vs. WW)/log_2_FC (WN vs. WW) was between 0.67–1.5. Thus, there were still 1766 DEGs mainly affected by both H_2_O_2_ and NaCl in HN vs. WW at this time, where 780 DEGs were up-regulated and 986 DEGs were down-regulated. All subsequent data analysis on HN vs. WW was mainly carried out for these 1766 DEGs (Supplementary Table 17).

Gene ontology (GO) enrichment analysis was performed on 1493 and 1766 DEGs according to the biological processes in HW vs. WW (Figure 2D) and HN vs. WW (Figure 2E), respectively. GO terms both in HW vs. WW and HN vs. WW mainly included “regulation of biological processes” (HW vs. WW, *P* = 1.67 × 10^–5^; HN vs. WW, *P* = 7.54 × 10^–8^), “response to chemicals” (HW vs. WW, *P* = 2.64 × 10^–20^; HN vs. WW, *P* = 5.98 × 10^–25^), “response to abiotic stresses” (HW vs. WW, *P* = 9.72 × 10^–19^; HN vs. WW, *P* = 1.39 × 10^–18^), and “regulation of transcription” (HW vs. WW, *P* = 1.56 × 10^–4^; HN vs. WW, *P* = 8.3 × 10^–8^). This implies that many genes up-regulated by individual H_2_O_2_ pretreatment alone or HN, or both, may be related to the enhanced salt tolerance of plants through their function in the abovementioned process. More interestingly, we found that the GO terms related to “cell cycle process” (*P* = 1.38 × 10^–19^) and “cell division” (*P* = 1.21 × 10^–9^) were specially enriched in HW vs. WW; therefore, we speculate that the up-regulation of these genes may affect plant growth to cope with the subsequent stresses.

To assess the major transcriptional dynamics associated with the responses to H_2_O_2_ pretreatment and/or both H_2_O_2_ and NaCl, we further clustered these DEGs from HW vs. WW and HN vs. WW into nine groups according to their expression trends under the four different combinations of treatments (Figures 2F,G). Of these clusters, we mainly focus on five clusters on the basis of their functional annotations and the expression profiles which were up-regulated either in HW vs. WW, HN vs. WW, or both. The K1 group clusters those genes which may be primed by H_2_O_2_ pretreatment and have up-regulated expression with subsequent salt stress. These genes are enriched in response to abiotic stimuli, illustrating their positive regulatory roles in increased plant salt tolerance. The genes in K2 were significantly up-regulated in HN vs. WW without obviously different expression in HW vs. WW and, so, they are enriched in oxidation–reduction processes, response to abiotic stimuli, and cell wall organization or biogenesis, which may mean that the expression of these genes is initiated during H_2_O_2_ pretreatment and mainly functions in subsequent salt stress. The K5 group comprises genes which mitigated the degree of up-regulation due to H_2_O_2_ pretreatment followed by NaCl, as compared with NaCl alone, which is characterized by an abundance of genes related to stress responses, especially osmotic stress and ABA, response to chemicals, and response to abiotic stimuli. The K7 group clusters those genes that were successively up-regulated by individual H_2_O_2_ and H_2_O_2_ plus NaCl, but the magnitude of the increase in the latter was less than in NaCl alone. Based on K5 and K7, we speculate that pretreatment with H_2_O_2_ can alleviate the oscillation of plant intracellular environment caused by subsequent NaCl exposure. The K8 group includes genes which were mainly up-regulated by H_2_O_2_ pretreatment, while only a few of these were up-regulated by combined H_2_O_2_ and NaCl treatment. Moreover, these genes are concentrated in cell cycle processes and cell division, implicating that after H_2_O_2_-induced expression, these genes may promote cell proliferation, resulting in plant growth under subsequent high-salt stress.

**FIGURE 3:**
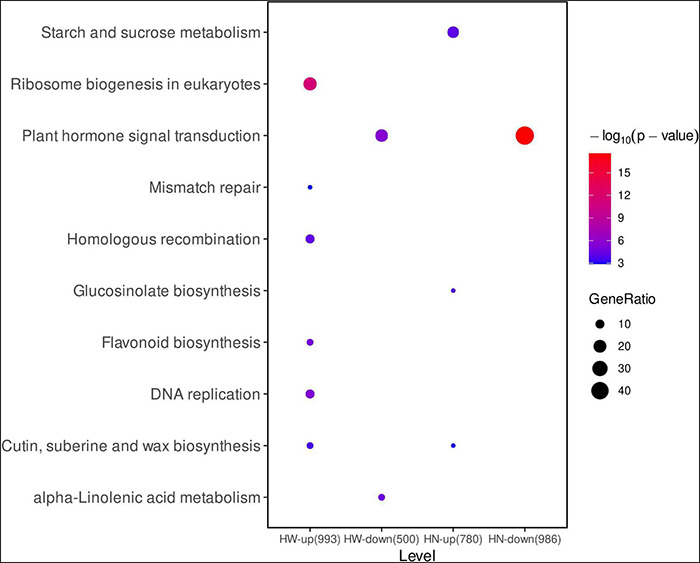
The kyoto encyclopedia of genes and genomes (KEGG) pathway of DEGs. The pathway names are provided on the vertical axis. The color of the dot represents *p* value and the size of dot represents gene ratio in each functional category. The rich level in the horizontal axis is the size of the point, which represents the number of DEGs, and the color of the dot represents the q value. HW-up (993), 993 up-regulated DEGs in HW vs. WW; HW-down (500), 500 down-regulated DEGs in HW vs. WW; HN-up (780), 780 up-regulated DEGs in HN vs. WW; HN-down (980), 980 down-regulated DEGs in HN vs. WW.

To identify the metabolic pathways in which the DEGs were involved and enriched, KEGG analysis was also performed. The results revealed that in HW vs. WW, 993 up-regulated genes were enriched in six pathways, including ribosome biogenesis in eukaryotes (ath03008, *P* = 3.46 × 10^–10^), DNA replication (ath03030, *P* = 0.00011), flavonoid biosynthesis (ath00941, *P* = 0.00030), homologous recombination (ath03440, *P* = 0.00082), cutin, suberine and wax biosynthesis (ath00073, *P* = 0.00275), and mismatch repair (ath03430, *P* = 0.00019); 500 down-regulated genes were assigned to plant hormone signal transduction (ath04075, *P* = 0.01571) and alpha-linolenic acid metabolism (ath00592, *P* = 0.000588). Likewise, in HN vs. WW, 780 up-regulated genes were enriched in starch and sucrose metabolism (ath00500, *P* = 0.00496), glucosinolate biosynthesis (ath00966, *P* = 0.00496), and cutin, suberine and wax biosynthesis (ath00073, *P* = 0.02513); while 986 down-regulated genes mainly participated in plant hormone signal transduction pathway (ath04075, *P* = 2.68 × 10^–18^) (Figure 3).

### H_2_O_2_-Pretreatment Activates Cell Cycle Process and Cell Division

**FIGURE 4:**
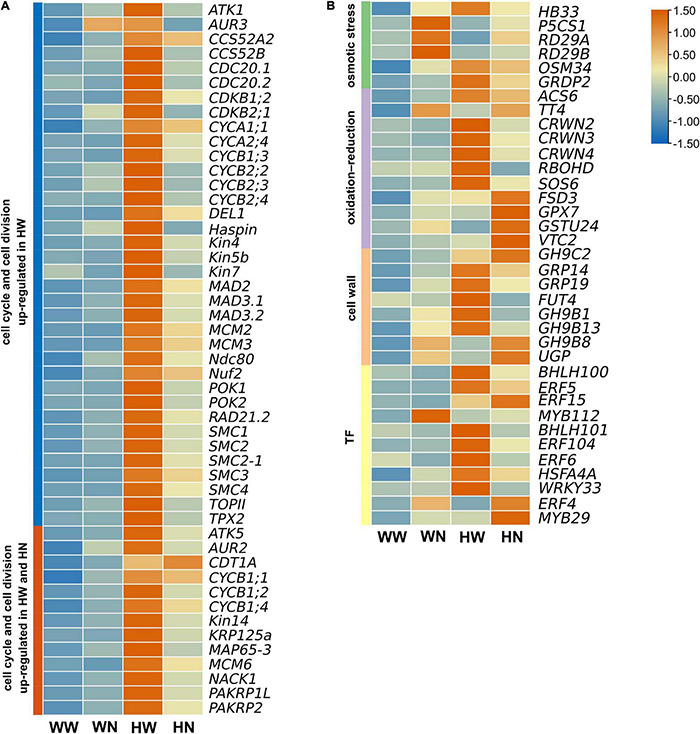
The expression patterns of selected differentially expressed genes (DEGs) represented as a heatmap. **(A)** Up-regulated DEGs related to the cell cycle and cell division only in HW vs. WW, or both in HW vs. WW and HN vs. WW. **(B)** Up-regulated DEGs related to salt stress only or both in HW vs. WW and HN vs. WW. Among osmotic stress responsive genes, *HB33* was up-regulated in HW vs. WW; *P5CS1*, *RD29A*, and *RD29B* were up-regulated in HN vs. WW. *BGLU6* and *GRDP2* were significantly up-regulated in HW vs. WW and HN vs. WW; Of DEGs involved in oxidation–reduction processes, *ACS6* and *TT4* were significantly up-regulated in HW vs. WW and HN vs. WW; *CRWN2*, *CRWN3*, *CRWN4*, *RBOHD*, and *SOS6* were up-regulated in HW vs. WW; *FSD3*, *GSTU24*, and *VTC2* were up-regulated in HN vs. WW; Among genes related to cell wall organization, *GH9C3* and *GRP14* were significantly up-regulated in HW vs. WW and HN vs. WW; *GRP19*, *FUT4*, *GH9B1*, and *GH9B13* were up-regulated in HW vs. WW; and *GH9B8* and *UGP1* were up-regulated in HN vs. WW; Of genes related to the transcription factors, *BHLH100*, *ERF5*, *ERF15*, and *WRKY38* were significantly up-regulated in HW vs. WW and HN vs. WW; *BHLH101*, *ERF104*, *ERF6*, *HSFA4A*, and *WRKY33* were up-regulated in HW vs. WW; and *ERF4* and *MYB29* were up-regulated in HN vs. WW. WW, pretreated with water and not salt-stressed; WN, pretreated with water and salt-stressed; HW, pretreated with H_2_O_2_ and not salt-stressed; HN, pretreated with H_2_O_2_ and salt-stressed. Heat map diagram of the log2FC, the red and blue colors specify up-and down-regulated expressions.

Further mining the transcriptome data, we found that after low-concentration H_2_O_2_ pretreatment of seedlings, a large proportion of genes related to the cell cycle and cell division were up-regulated, and most were significantly induced only under HW vs. WW (Figure 4A, Table 1, and Supplementary Table 18). Even though the expression levels of a few genes were increased under HW vs. WW and HN vs. WW, the extent of increase in the former was higher than in the latter (Figure 4A, Tables 1, 2, and Supplementary Tables 18, 20). Among these are sixteen core cell cycle genes, including two A-type cyclins (*CYCA1;1, CYCA2;4*), seven B-type cyclins (*CYCB1;1, CYCB1;2, CYCB1;3, CYCB1;4, CYCB2;2, CYCB2;3, CYCB2;4*), two plant-specific B-type *CDKs* (*CDKB1;2; CDKB2;1*) and upstream regulator *DEL1* and its target *CDT1A*, and minichromosome maintenance genes (*MCM2, MCM3, MCM6*). B-type *CDKs* are plant-specific and are divided into two subtypes: *CDKB1* and *CDKB2. CDKB1* is activated by A2-type and all B-type cyclins and functions in the late S-to-M phase, while B2-type *CDKs* exclusively associate with B1-type cyclins and have transcript levels peaking late in the M phase (Van Leene et al., 2010). In our data, the increased transcript levels of both *CDKB1;2, CDKB2;1* and their corresponding partners *CYCB2;4, CYCB1;1* implied that the CDKB1;2/CYCB2;4 and CDKB2;1/CYCB1;1 complex may promote cell cycle progression through late S-to-M or M phases (Van Leene et al., 2010).

**TABLE 1:** Expression levels of DEGs from different biological processes in HW vs. WW.

Gene ID	Log_2_FC	*P*-value	Annotation
**Signal transduction**			
*AT3G17840*	1.261703332	4.29373E-17	*RLK902*
*AT3G45640*	1.334456738	4.26075E-36	*MPK3*
*AT5G01820*	1.109435057	1.39871E-22	*CIPK14*
**Response to cell cycle progress**			
*AT4G37490*	1.750432008	0.001885814	*CYCB1;1*
*AT4G35620*	1.763118962	1.53352E-05	*CYCB2;2*
*AT5G51600*	2.642908158	1.48104E-16	*MAP65-3*
*AT1G03780*	2.432668374	1.31158E-09	*TPX2*
*AT5G62410*	1.823633283	3.30199E-10	*SMC2*
**Response to osmotic stress**			
*AT1G60270*	1.323197896	0.000191456	*BGLU6*
*AT4G11650*	2.113939835	7.81E-06	*OSM34*
*AT5G24780*	3.299359332	4.734E-144	*VSP1*
*AT1G75240*	1.415108543	1.91168E-06	*HB33*
*AT4G37900*	2.605918804	7.81889E-17	*GRDP2*
**Response to oxidation-reduction process**			
*AT4G11280*	1.536109176	1.20768E-30	*ACS6*
*AT5G13930*	2.248563722	4.07287E-52	*TT4*
*AT1G02730*	1.959872057	1.7911E-25	*SOS6*
*AT5G47910*	1.011550101	5.92512E-12	*RBOHD*
*AT1G13220*	1.354413099	7.05061E-05	*CRWN2*
*AT1G68790*	1.099191276	0.000254254	*CRWN3*
*AT5G65770*	1.12323409	2.21483E-05	*CRWN4*
*AT5G16960*	3.065392853	0.000105708	*Oxidoreductase*
**Response to cell wall organizations**			
*AT1G19940*	1.2261646	0.011564313	*GH9B5*
*AT1G64390*	1.072218801	5.01998E-08	*GH9C2*
*AT5G07550*	8.004993817	3.56984E-12	*GRP19*
*AT5G07510*	3.689585073	1.93056E-06	*GRP14*
*AT2G15390*	1.19716344	1.21643E-18	*FUT4*
*AT1G70710*	1.048101372	1.65661E-09	*GH9B1*
*AT4G02290*	2.700819045	1.90368E-18	*GH9B13*
**Transcription factors**			
*AT1G48000*	1.569497531	0.002033524	*MYB112*
*AT2G31230*	1.201997627	0.008076579	*ERF15*
*AT4G17490*	1.197983855	1.3033E-06	*ERF6*
*AT5G47230*	2.395906495	2.1177E-07	*ERF5*
*AT5G61600*	1.954431983	2.9311E-12	*ERF104*
*AT2G26150*	1.451800124	9.54612E-06	*HSFA2*
*AT4G18880*	1.241326498	2.06376E-21	*HSFA4A*
*AT2G41240*	2.425281785	5.34598E-10	*BHLH100*
*AT5G04150*	2.182273047	6.61157E-12	*BHLH101*
*AT2G38470*	2.05231484	6.12108E-76	*WRKY33*
*AT2G46400*	2.351716545	3.99027E-24	*WRKY46*

**TABLE 2:** Expression levels of DEGs from different biological processes in HN vs. WW.

Gene ID	Log_2_FC	*P*-value	Annotation
**Signal transduction**			
*AT3G17840*	1.147633728	6.42534E-18	*RLK902*
*AT2G38490*	2.403252429	0.00190412	*CIPK22*
*AT2G01505*	2.337022049	6.25275E-10	*CLE16*
*AT1G16540*	1.007043314	1.07322E-05	*ABA3*
*AT5G57050*	1.3133036424	6.07E-26	*ABI2*
**Response to cell cycle progress**			
*AT4G37490*	1.580586106	0.006747561	*CYCB1;1*
*AT5G06150*	1.386218026	4.42546E-06	*CYCB1;2*
*AT2G31270*	1.882548482	1.03224E-10	*CDT1A*
*AT4G14330*	1.945203044	2.51984E-06	*PAKRP2*
*AT3G23670*	1.630135308	2.62069E-05	*PAKRP1L*
**Response to osmotic stress**			
*AT5G52310*	1.29017064	4.34762E-63	*RD29A*
*AT5G52300*	1.478185515	4.17866E-12	*RD29B*
*AT1G60270*	1.503569655	6.36039E-06	*BGLU6*
*AT4G11650*	2.37714146	0.000217914	*OSM34*
*AT2G39800*	1.360964115	4.84828E-82	*P5CS1*
*AT1G35910*	2.534489463	0.000065004	*TPPD*
*AT4G37900*	1.899733107	9.70682E-05	*GRDP2*
**Response to oxidation-reduction process**			
*AT4G11280*	1.2403011	1.09258E-24	*ACS6*
*AT5G13930*	3.315570351	6.10798E-72	*TT4*
*AT4G31870*	2.580344735	3.14048E-06	*GPX7*
*AT5G23310*	1.454724899	1.30858E-22	*FSD3*
*AT4G26850*	1.010302971	3.01913E-42	*VTC2*
*AT1G17170*	1.449349647	0.007255035	*GSTU24*
*AT5G16960*	3.406965131	4.14553E-06	*Oxidoreductase*
**Response to cell wall organizations**			
*AT1G19940*	1.489276871	0.002939534	*GH9B5*
*AT1G64390*	1.491245104	3.22699E-55	*GH9C2*
*AT3G03250*	1.010628344	2.29E-33	*UGP1*
*AT2G32990*	2.117703788	4.25088E-36	*GH9B8*
*AT5G07510*	3.068711839	1.52731E-07	*GRP14*
**Transcription factors**			
*AT1G48000*	2.021916518	7.61648E-06	*MYB112*
*AT5G47230*	1.641220877	9.08404E-15	*ERF5*
*AT2G31230*	1.712136322	6.87902E-08	*ERF15*
*AT3G15210*	1.035087685	2.64451E-11	*ERF4*
*AT2G41240*	1.022260604	2.14167E-06	*BHLH100*
*AT5G07690*	1.817904344	8.14544E-35	*MYB29*
*AT5G43840*	2.5429776	0.000595319	*HSFA6A*

Besides CDKs and their cyclin partners, E2F transcription factors also belong to the core cell cycle machinery. Upon H_2_O_2_ pretreatment, the atypical *E2F DP-E2F-like 1* (*DEL1*) was up-regulated, indicating that *DEL1* may enhance cell proliferation by repressing the transcription of *CCS52A2*, which is required for endocycle onset (Lammens et al., 2008). *DEL1* can also restrain the stress-induced switch from mitosis to the endocycle in dividing cells exposed to osmotic stress (Cookson et al., 2006). Contradictory with *DEL1* inhibiting *CCS52A2*, *CCS52A2* was also markedly up-regulated only under HW; this inconsistency may have been due to using transcriptomic data from whole shoots of Arabidopsis instead of defined cells. Besides *CCS52A2*, other anaphase-promoting complex/cyclosome (APC/C) coactivators such as CCS52B and CDC20 (CDC20.1, CDC20.2) were significantly elevated in transcripts under HW compared to WW, and this may be responsible for facilitating the switch from mitosis to endoreduplication through targeting of mitotic cyclins for destruction, thus inactivating cyclin-dependent kinase (CDK) (Kevei et al., 2011; Yang et al., 2017). CDKs/cyclins and their regulators DEL1, CCS52A2, CCS52B, CDC20.1, and CDC20.2 coordinate to balance cell proliferation and cell differentiation/expansion and, thus, balance plant growth and development.

Furthermore, H_2_O_2_ pretreatment also induced the expression of *CDT1A* and minichromosome maintenance genes (*MCM2, MCM3, MCM6*), where only *CDT1A* and *MCM6* were differentially expressed under HW and HN, while only *MCM2* and *MCM3* were differentially expressed under HW (Figure 4A). CDT1A, as a DNA replication licensing factor, can recruit the MCM complex to form the components of the pre-replicative complex at the G1 phase (Nishitani et al., 2001). Therefore, high transcript levels of these genes facilitate activation of the replication origin, which can ensure that genomic DNA is replicated completely and accurately only once during the S phase in a single cell cycle (Tuteja et al., 2011). Meanwhile, according to previous research results, the high H_2_O_2_-induced expression of *MCM6* can presumably confer plant salt tolerance by preserving normal DNA replication under salinity stress conditions (Dang et al., 2011).

Except for the abovementioned cell cycle components, low levels of H_2_O_2_ also increased transcription of a large number of genes encoding spindle assembly factors. These include genes for mitosis kinases (AUR2, AUR3, and AtHaspin); microtubule-associated proteins (MAPs), including TPX2, MAP65-3, and members of the kinesin superfamily (Kin4/chromokinesin, Kin5, Kin7, Kin12 and Kin14 families); chromosome organization proteins (SMC1, SMC2, SMC3, SMC4, RAD21.2, and TOPII); kinetochore complex (Ndc80 and Nuf2); and spindle assembly checkpoint complex (Mad2, Mad3.1, and Mad3.2). Among these, AtHaspin can activate AUR3 and promote its centromeric localization on chromosomes by phosphorylating histone H3 at Thr3. Then, AtHaspin and AUR3 together regulate proper chromosome alignment in the spindle during prometaphase/metaphase and chromosome segregation (Kozgunova et al., 2016). In this process, the cohesin complex, containing SMC1, SMC3, and RAD21, can contribute to chromosome alignment, while TOPII can release these cohesins from chromosomes to allow for chromosome segregation (Higgins, 2010; Kamenz and Hauf, 2017). In addition, the condensin complex, comprising SMC2 and SMC4, also ensures chromosome condensation and proper segregation (Wang H. et al., 2019). Ndc80 and Nuf2, as components of the kinetochore complex, are localized at the outer kinetochore, connecting spindle fibers to the kinetochore as well as mediating chromosome segregation during cell division (Shin et al., 2018). In mitosis, the SAC core proteins Mad2 and Mad3.2 are recruited to the kinetochore that is unattached to the spindle; then, Mad2 and Mad3.2 together with Mad3.1 may bind CDC20 to form the mitotic checkpoint complex (MCC) to inhibit the activity of APC/C (Komaki and Schnittger, 2017). Until the kinetochore is correctly attached to the spindle, CDC20 is released, which then activates APC/C for the removal of cohesin, thus promoting entry into anaphase (Singh et al., 2014). Moreover, AUR3 is present at kinetochores and is involved in kinetochore assembly during mitosis (Lermontova et al., 2015). Therefore, during exposure to H_2_O_2_ or combined H_2_O_2_ and NaCl treatment, all these up-regulated genes may coordinate to control proper condensation and segregation of chromosomes for successful cell division.

AUR2 is another member of the Arabidopsis Aurora kinase family, which is associated with spindle assembly, phragmoplast organization, and cell plate orientation during mitotic division (Demidov et al., 2014). In this process, AUR2 activity may be controlled by its upstream regulators AtHaspin (Kozgunova et al., 2016) and TPX2 (Petrovska et al., 2012). TPX2 is a MAP with multiple functions in microtubule organization, and can activate and phosphorylate AUR2. The TPX2–AUR2 complex can colocalize on spindle microtubules during mitosis and thereby control cell division (Petrovska et al., 2012).

Besides TPX2, many other MAPs regulate microtubule dynamics for the proper formation of different MT arrays during the cell cycle. AtMAP65-3 begins to accumulate at the narrow midline of the spindle at metaphase and is involved in antiparallel MT bundling at the phragmoplast midline at telophase. Similar to AtMAP65-3, kinesin-5 interdigitates microtubules at both spindle and phragmoplast midline (Bannigan et al., 2007). The kinesin-7 family member NACK1 participates in phragmoplast organization by recruiting MAPKKK (ANP) to the phragmoplast midline and activating the MAP kinase cascade during the late mitosis phase, which is critical for cell plate formation (Sasabe et al., 2015). The kinesin-12 family members POK1 and POK2 are important for PPB function (Rasmussen et al., 2011), and PAKRP1 and PAKRP1L are involved in MT interdigitation at the phragmoplast midline (Lee et al., 2007). ATK1 and ATK5 are two minus-end-directed kinesin-14s which are essential in spindle assembly and function (Ambrose and Cyr, 2007). Therefore, the H_2_O_2_-induced expression of all these *MAP* genes contributes to the assembly of microtubule arrays and the progression of cell division.

The abovementioned cell cycle genes are associated with mitotic cell cycle, chromatin dynamics, and microtubule-related processes in promoting cell proliferation and maintaining the normal structure of chromosomes. As cell proliferation and cell expansion are the main driving forces in leaf growth, the up-regulation of these genes may maintain plant growth in cope with subsequent stresses; however, the mechanisms by which low levels of H_2_O_2_ promote plant cell cycle progression and growth remain unclear.

### Differentially Expressed Genes Associated With Osmotic Stress

Plants first adopt a series of molecular mechanisms in response to osmotic stress when exposed to high salinity, such as regulating the expression of many genes involved in stomatal closure and synthesizing osmotically protective substances (Feng et al., 2016).

In HW vs. WW, some of the identified DEGs were osmotic stress-responsive (Table 1 and Supplementary Tables 18, 19), but relatively few of these types of genes were up-regulated. Of these, *HB33* was induced mainly by H_2_O_2_, while the transcript increases under HN and WN were not distinctly different from WW (Figure 4B). Some studies have shown that HB33 is a positive regulator in ABA, mediating plant growth and development as well as response to different abiotic stresses, such as osmotic stress (Wang et al., 2011). In HN vs. WW, more osmotic stress-responsive DEGs were up-regulated (Table 2 and Supplementary Tables 20, 21). *RD29A*, *RD29B*, and *P5CS1* are typical representatives, but the magnitude of their expression increases were less than in WN vs. WW (Figure 4B). We speculate that pretreatment with H_2_O_2_ perhaps mitigates the osmotic stress caused by subsequent salt stress. *RD29A* and *RD29B* are osmotic stress-related marker genes, and their encoding proteins RD29A and RD29B act as protective molecules in response to osmotic stress. *P5CS1* encodes a key enzyme in proline biosynthesis and promotes proline accumulation to confer plant osmotic stress resistance (Feng et al., 2016). Comparing the DEGs in HW vs. WW with those in HN vs. WW, we found that *OSM34* and *GRDP2* were significantly up-regulated under both conditions (Tables 1, 2); however, their expression patterns were different. The expression level of the former in HN was higher than that in HW, while for the latter, the converse was observed (Figure 4B). *Osmotin34* (*OSM34*) encodes osmotin to combat osmotic stress (Sharma et al., 2013), and *AtGRDP2* encodes a short glycine-rich domain protein which may improve the growth of plants under osmotic stress (Ortega-Amaro et al., 2014).

### Differentially Expressed Genes Associated With Oxidation-Reduction Process

To investigate which genes or biological processes are involved in the H_2_O_2_-primed oxidative stress tolerance of plants, we performed GO analysis on all DEGs in HW vs. WW and HN vs. WW, and found that some DEGs which may protect plants from damage during subsequent salt stress that were activated by H_2_O_2_ (Supplementary Tables 18–21).

Comparing the DEGs in HW vs. WW with those in HN vs. WW, we found that *ACS6* and *TT4* were jointly up-regulated under both conditions (Tables 1, 2); however, their expression patterns were distinct, with *ACS6* expression higher in HW than in HN, whereas *TT4* expression increased progressively with HW and HN (Figure 4B). *ACS6* is one of the most important genes in ethylene biosynthesis, controlling the level of ethylene. Datta et al. (2015) reported that *ACS6* was significantly up-regulated during the glutathione–ethylene interaction in response to salt stress. Our transcriptomic data implies that H_2_O_2_ pretreatment may elevate ethylene production by *ACS6* transcription increase to activate the ROS-detoxifying system in defending against subsequent salt stress. The expression pattern of *ACS6* further confirmed that ethylene may participate in H_2_O_2_-primed redox balance reconstruction under salt stress. *TT4* encodes chalcone synthase (CHS), a key enzyme involved in the biosynthesis of flavonoids. It has been reported that the up-regulation of *TT4* led to an increase in anthocyanin synthesis. Anthocyanins can function as antioxidants, helping plants to scavenge ROS and maintaining redox homeostasis during salt stress. The DEGs only up-regulated in HW vs. WW, such as *CRWN2*, *CRWN3*, *CRWN4*, *SOS6*, and *RBOHD*, were all clustered to K8 (Figures 2F, 4B and Table 1). *CRWNs* constitute a small gene family containing only *CRWN1–4* members, three of which were detected in our transcription data. CRWN proteins have been reported to maintain the size and morphology of the nucleus in order to promote the normal growth of plants (Wang et al., 2013). We speculate that H_2_O_2_ induces the expression of *CRWN2*, *CRWN3*, and *CRWN4*, which may be positive regulators of oxidative stress tolerance, inhibiting ROS production and DNA damage during subsequent salt stress (Wang Q. et al., 2019). *RBOHD* is a key member of the *RBOHs* family, where RBOH-mediated spatiotemporal control of ROS production is required for appropriate cell elongation. We consider that H_2_O_2_ pretreatment promoted moderate expression of *RBOHD*, thereby promoting the growth and development of plants and improving salt tolerance (Marino et al., 2012; Suzuki et al., 2012). *SOS6* has an important role in osmotic stress tolerance and may be involved in the regulation of ROS levels under oxidative stress (Yang et al., 2016). The DEGs only in HN vs. WW include *VTC2*, *FSD3*, *GPX7*, and *GSTUs*, all of which were up-regulated (Figure 4B and Table 2). *VTC2* encodes GDP-L-galactose phosphorylase, catalyzing the conversion of GDP-L-Gal into L-Gal, which is considered to be a committed step in ascorbate biosynthesis (Koffler et al., 2014). Ascorbate, as a relatively abundant small-molecule antioxidant in plants, can detoxify ROS throughout the cell. FeSOD is one of the three major classes of SOD. Overexpression of *FSD3* results in great tolerance to oxidative stress through scavenging of ROS (Myouga et al., 2008). GPXs are important ROS scavengers due to their broad substrate specificity and high affinity for H_2_O_2_. The up-regulated expression of *GPX7* has been shown to be important for maintenance of redox balance in the cell (Chang et al., 2009). Glutathione S-transferases (GSTs) protect plants from oxidative damage and enhance the antioxidant capacity of plants. We also found that several *GSTUs* were up-regulated, such as *GSTU24*.

### Differentially Expressed Genes Associated With Cell Wall Organizations

The plant cell wall is the first defense against external environmental stresses. To check whether H_2_O_2_ pretreatment invoked the expression of genes encoding for cell wall components, we further analyzed the transcription data and found that, based on GO analysis, some genes could be primed by H_2_O_2_ pretreatment (Supplementary Tables 18, 19), whereas some genes were regulated by subsequent salt stress (Supplementary Tables 20, 21). These genes are involved in the regulation of the synthesis of various components of the cell wall, causing the cell wall to harden. The formation of a physical barrier protects plant cells from further dehydration and death under salt stress, thereby resisting salt stress.

The identical DEGs in HW vs. WW and HN vs. WW, such as *AtGH9C2* and *GRP14*, were up-regulated (Figure 4B and Tables 1, 2). AtGH9C2 is a class C endo-1, 4-β-glucanase (cellulase). Some studies have found that such endoglucanases affect cell wall development by promoting cell wall crystallization processes (Glass et al., 2015). GRP14 is an important structural protein which is widely found in plant cell walls, and the expression of *GRP14* helps in cell wall remodeling when plants are exposed to salt stress (Le Gall et al., 2015). The DEGs up-regulated only in HW vs. WW, such as *AtGH9B1*, *AtGH9B13*, *GRP19*, and *FUT4*, were clustered to K7 (Figures 2F, 4B and Table 1). AtGH9B1 and AtGH9B13 are both class B endoglucanases that play an important role in cell wall relaxation during cell growth and expansion (Tsabary et al., 2003; Urbanowicz et al., 2007). GRP19 is a glycine-rich protein with similar function to GRP14 (Le Gall et al., 2015). FUT4 is an arabinogalactan (AG)-specific fructosyltransferase (FUT), which is responsible for the fructosylation of proteins glycosylated with arabinogalactan (AGPs) in leaves, which maintains proper cell expansion and root growth under salt stress conditions (Tryfona et al., 2014).

The DEGs up-regulated only in HN vs. WW, such as *AtGH9B8* and *UGP*, were the representatives of cluster K2 (Figures 2F, 4B and Table 2). Like AtGH9B1 and AtGH9B13, AtGH9B8 also belongs to class B endoglucanases and is involved in cell wall relaxation during cell growth and expansion (Mele et al., 2003; Urbanowicz et al., 2007; Tryfona et al., 2014). The up-regulated expression of *UGP1* can promote the biosynthesis of the cell wall, maintaining plant growth under salt stress and, therefore, increasing the salt tolerance of plants, after H_2_O_2_ pretreatment.

### Differentially Expressed Genes Associated With Transcription Factors

Following H_2_O_2_ pretreatment, some transcription factors were accumulated in plants to defend against subsequent high-salt stress. After GO analysis, we identified some transcription factors encoding genes in HW vs. WW (Supplementary Tables 18, 19) and HN vs. WW (Supplementary Tables 20, 21), respectively. The gene number in the former accounts for a large proportion of the DEGs, with significantly more than the latter. These transcription factors can be divided into five categories, belonging to the ERF, MYB, WRKY, HSFA, and bHLH families.

The identical DEGs up-regulated in HW vs. WW and HN vs. WW included *MYB112*, *ERF5*, *ERF15*, and *bHLH100* (Tables 1, 2); however, the expression levels of *bHLH100* and *ERF5* were higher in HW than in HN, while the converse was the case for *ERF15* and *MYB112* (Figure 4B). *MYB112* is a member of the *R2R3 MYBs*. The up-regulated expression of *MYB112* promotes the accumulation of anthocyanins, which can respond to different abiotic stresses, including oxidative stress, osmotic stress, and high-salt stress (Lotkowska et al., 2015). The DEGs up-regulated only in HW vs. WW, including *WRKY33*, *ERF6*, *ERF104*, *bHLH101*, and *HSFA4A*, clustered to K8 (Figures 2F, 4B and Table 1). Some studies have shown that *WRKY33*, *ERF6*, and *ERF104* can regulate the expression of salt-tolerant genes in different signaling pathways, and that the overexpression of these genes can increase salt tolerance in plants (Jiang and Deyholos, 2009; Vogel et al., 2014; Van den Broeck et al., 2017). *bHLH101* can increase the oxidative stress tolerance of plants (Noshi et al., 2018). *HSFA4A* encodes a member of heat stress transcription factors (Hsfs), certain members of which have been shown to function as ROS-dependent redox sensors, controlling gene expression during oxidative stress. Although we do not know whether HSFA4A is such a redox sensor, there has been evidence showing that it plays key roles in a variety of stress signaling pathways, and its overexpression enhances a variety of stress tolerances, including salt stress, osmotic stress, oxidative stress, and heavy metal stress (Perez-Salamo et al., 2014; Lin et al., 2018).

The DEGs up-regulated only in HN vs. WW include *ERF4* and *MYB29* (Figure 4B and Table 2). *ERF4* are important molecules in the signaling pathways of ethylene and jasmonic acid, regulating the expression of a large number of genes involved in many plant defense mechanisms. Overexpression of *ERF4* has been shown to increase salt and drought stress tolerance in plants (Seo et al., 2010).

### Confirmation of RNA-Seq Data by RT-qPCR

**FIGURE 5:**
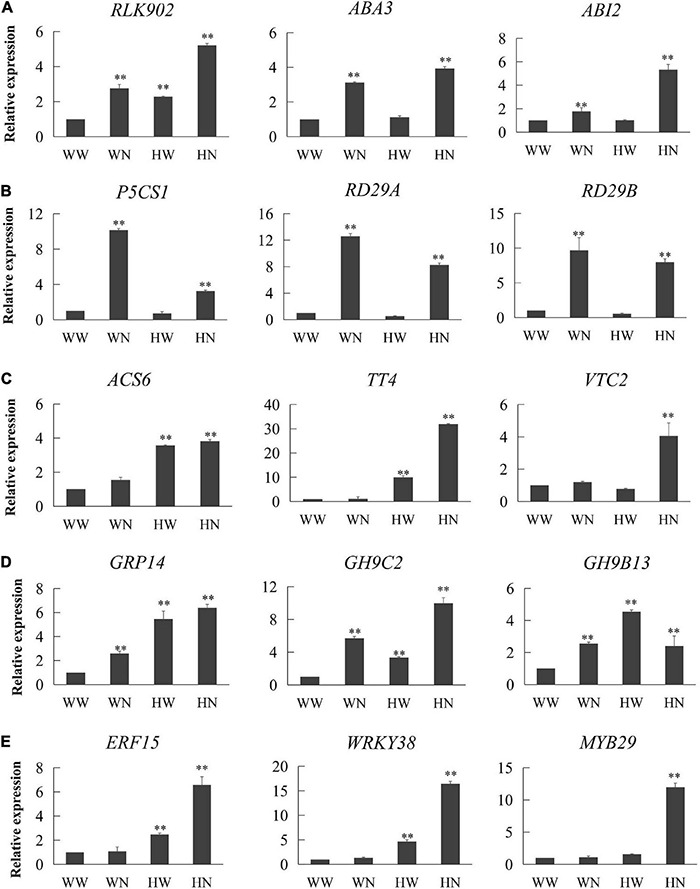
Confirmation of transcriptional changes by RT-qPCR. We selected 15 genes from the transcriptome to detect their expression trends: **(A)** genes involved in signal transduction, **(B)** genes involved in responding to osmotic stress, **(C)** genes involved in response to oxidation–reduction process, **(D)** genes involved in cell wall organization, and **(E)** genes related to transcription factors. The expression trends of these genes in the transcriptome are given in Tables 1, 2. RT-qPCR results are presented in the form of 2^–△△*CT*^. Three biological replicates per experiment. Data are represented as means ± SD. * and ** represented significantly and very significantly different at *P* < 0.05 and *P* < 0.01, respectively, estimated with one-way ANOVA following SPSS. WW, pretreated with water and not salt-stressed; WN, pretreated with water and salt-stressed; HW, pretreated with H_2_O_2_ and not salt-stressed; HN, pretreated with H_2_O_2_ and salt-stressed.

To further validate whether the expression of DEGs was induced by only H_2_O_2_ pretreatment or both H_2_O_2_ pretreatment and subsequent salt stress, we selected 15 genes involved in signal transduction (Figure 5A), response to osmotic stress (Figure 5B), response to oxidation–reduction process (Figure 5C), cell wall organization (Figure 5D), and transcription factors (Figure 5E) for RT-qPCR. It was verified that the expression trends of these genes tested by RT-qPCR were highly consistent with the transcriptome data; therefore, the conclusions obtained from the transcriptome analysis are reliable. Primer information is presented in Supplementary Table 1.

### Influence of H_2_O_2_ Pretreatment on Antioxidative Enzyme Activities of Seedlings

Salinity-induced oxidative stress in plants is associated with ROS overproduction. Through transcriptome analysis, we found some genes involved in the scavenging of ROS, such as *FSD3* and *GPX7*.

**FIGURE 6:**
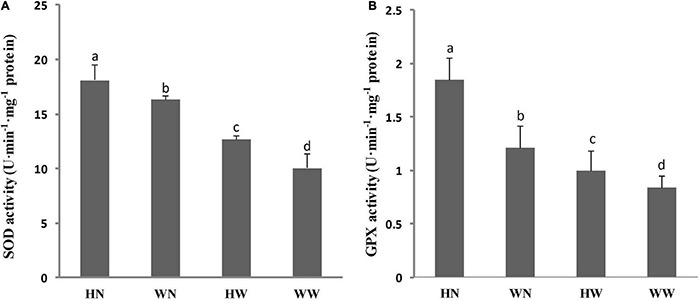
Influence of H_2_O_2_ pretreatment on SOD **(A)**, and GPX **(B)** activities in Arabidopsis leaves under different treatments. Three biological replicates per experiment. Data are represented as means ± SD. Means for each treatment that do not share a common letter are significantly different at *P* < 0.05, estimated with one-way ANOVA following SPSS. WW, pretreated with water and not salt-stressed; WN, pretreated with water and salt-stressed; HW, pretreated with H_2_O_2_ and not salt-stressed; HN, pretreated with H_2_O_2_ and salt-stressed.

The total SOD, and GPX activities in leaves are shown in Figure 6. The results show that the SOD activities in the HN and WN groups were greatly higher than those in the HW and control (WW) groups, which exhibit consistency between SOD activity and *FSD3* expression. Under stress treatment, SOD activity in the HN group increased greatly compared with that in the WN group (Figure 6A). GPX activities in the HN group were significantly higher than in the WN group and, similarly, markedly higher in the HW group than in the WW group (Figure 6B). These results for GPX activity coincided with *GPX7* expression.

## Discussion

Salt stress results in osmotic stress and oxidative stress, which limit plant growth and development and subsequently reduce crop yields. Plants can adapt to stressful environments through many physiological and molecular mechanisms, and plant resistance can be improved by many methods.

Seedling treatment with inorganic and organic agents greatly reduces the detrimental effects of stress and enhances essential nutrient content (Qiu et al., 2014; Cantabella et al., 2017; Ghassemi-Golezani and Farhangi-Abriz, 2018; Silva et al., 2020; Zahedi et al., 2021). In our present work, the pretreatment of seedlings with H_2_O_2_ increased the fresh and dry weights of salinity-treated seedlings.

H_2_O_2_ pretreatment helped seedlings to reduce the accumulation of Na^+^ and improve the K^+^ content and K^+^/Na^+^ ratio. Increased tissue K^+^ content and K^+^/Na^+^ ratio are important for retaining metabolic activities and, therefore, have been taken as valid physiological criteria for salt tolerance.

The stability of biological membranes has also been used as a screening tool to assess salt stress effects (Farooq and Azam, 2006). Seedling pretreatment with H_2_O_2_ reduced RMP in this study, although the change was not much different between the HW group and control. Lipid peroxidation of the plasma membrane is an important indicator of oxidative membrane damage induced by salt (Guo et al., 2017; Ma et al., 2017). Lipid peroxidation will eventually engender MDA, which can cause serious damage to cells. MDA has strong crosslinking properties and can bind with phosphatidyl ethanolamine, nucleic acid, and some amino acids, thereby producing lipofuscin-like pigments. Previous studies have found that the accumulation of MDA exhibited a positive correlation with an increase in plasma membrane permeability. Our experimental data showed that under salt stress conditions, the MDA content in H_2_O_2_-pretreated Arabidopsis seedlings was obviously lower than that in water-pretreated seedlings, indicating that H_2_O_2_ pretreatment can effectively alleviate the salt stress damage to the integrity and stability of the plant cell membrane. Therefore, the protective role of H_2_O_2_ involves improved tolerance of Arabidopsis seedlings to salt stress and maintenance of their growth during salt stress (De Azevedo Neto et al., 2005; Fedina et al., 2009; Wang et al., 2010; Gondim et al., 2012). Our research not only contributes to a better understanding of stress tolerance mechanisms in plants but is also of considerable value in developing effective methods for crop protection against environmental stresses during agricultural practice (Wahid et al., 2007).

To clarify the mechanisms implied in the physiological changes, we subsequently performed transcriptomic work to mine the gene expression patterns under different treatments (WW, WN, HW, and HN). Interestingly, a large number of genes involved in cell cycle control were up-regulated only or mainly in HW, including the core cell cycle genes (*CYCs*, *CDKB1;2, CDKB2;1*, their upstream regulator *DEL1* and its target *CDT1A*, as well as *MCM2*, *MCM3*, and *MCM6*) and spindle assembly factor-encoding genes (mitosis kinases, MAPs, kinesin superfamily, chromosome organization proteins, and kinetochore complex). These cell cycle genes can promote cell proliferation and maintain the normal structure of chromosomes. Due to cell proliferation and cell expansion being the main driving forces for plant growth, we speculate that the up-regulation of these genes may enable maintenance of plant growth in coping with the subsequent stresses, which has been exemplified in many studies; for example, *CYCB1;1* and *CYCB2;2* overexpression in rice plants led to the accelerated growth of plants (Lee et al., 2003) and the potential contribution of the overexpression of *MCM6* to the normal progression of DNA replication under salinity stress conditions and, thus, conferring salt tolerance in transgenic tobacco (Dang et al., 2011). Therefore, these findings provide strong supports for our conclusion based on our data: that H_2_O_2_ pretreatment enhanced Arabidopsis plant growth and salt tolerance. However, the mechanisms by which low levels of H_2_O_2_ promote plant cell cycle progression and growth remain unclear.

In various types of animal cells, it is already well-accepted that low levels of H_2_O_2_ can accelerate cell proliferation, perhaps by controlling the redox-dependent expression of D- and B-type cyclins (mainly D1 and B1 cyclins) and, thus, promoting G0/G1-to-S or S-to-G2 and -M cell cycle phase transitions (Burch and Heintz, 2005). In plants, D-type cyclins are also important regulators of G0/G1-to-S cell cycle phase transition, and ROS together with auxin may also play a role in the cell cycle activation of differentiated leaf cells by CDKA1 activation and acceleration of cell cycle re-entry (G0-to-G1) (Feher et al., 2008). Yu et al. (2016) reported that 25 μM H_2_O_2_ treatment increased the rate of cell division in the quiescent center of wild-type Arabidopsis root. In our work, H_2_O_2_ pretreatment enhanced the expression of many genes encoding B- rather than D-type cyclins. As a result, we propose that H_2_O_2_ can also expedite Arabidopsis cell proliferation mainly through promoting the S-to-M cell cycle phase transition. These cell cycle genes might contribute to the good performance of Arabidopsis plants under salt stress after H_2_O_2_ pretreatment. However, this speculation needs more in-depth research for confirmation.

Once a low concentration of H_2_O_2_ is applied to the blade surface, it can act as a signaling molecule, being sensed and delivered by certain proteins, including HSFA4A of the HSFA family (Miller and Mittler, 2006). The communication between cells and the extracellular environment is largely controlled by RLKs in plants (He and Wu, 2016). Our transcription data show that *HSFA4A* and *RLK902* were significantly up-regulated in HW, which implies that *HSFA4A* and *RLK902* may act as H_2_O_2_ sensors and transmit the H_2_O_2_ signal to activate transcription factors, including those in the WRKY, ERF, MYB, HSFA, and bHLH families. Then, these transcription factors regulate a series of downstream stress-responsive genes, ultimately improving plant growth and salt tolerance (Miller and Mittler, 2006; He and Wu, 2016).

As a key signaling molecule, H_2_O_2_ also connects the signaling pathways of multiple phytohormones; this connection was first found between H_2_O_2_ and ethylene. Besides ethylene, other key phytohormones such as abscisic acid, jasmonates (JAs), ethylene and salicylic acid are also closely related to H_2_O_2_. All of these phytohormones employ H_2_O_2_ in their signaling cascades, either upstream or downstream, to orchestrate plant growth, development, and stress responses (Saxena et al., 2016). In our work, we detected several up-regulated genes involved in hormone synthesis or related signaling pathways. Among them, *ABA1* and *ACS6* play a role in the first steps of ABA and ET biosynthesis, whereas *ABA3*, *ABI2*, *ERF1*, *ERF4*, *ERF6*, *ERF106*, *MYB51, WRKY70*, and *VSP1* are involved in ABA, ET, or JA signal transduction. In addition, *RBOHD*, an important member of the RBOH family, was induced by H_2_O_2_ pretreatment. RBOHs have been recognized as important targets in the response of phytohormones and H_2_O_2_ to various environmental cues (Yao et al., 2017). Recently, H_2_O_2_ generated by RBOHs was found to be essential for the maintenance of acquired thermotolerance during recovery after acclimation (Sun et al., 2018). Accordingly, plants primed with H_2_O_2_ or with a higher basal level of H_2_O_2_ formation will exhibit enhanced resistance to stressors (Ellouzi et al., 2017).

Base on KEGG pathway analysis, more down-regulated genes were enriched in phytohormone signal transduction. Of them, many JA biosynthesis related genes, such as *LOXs* (*LOX3* and *LOX4*), *AOCs* (*AOC1* and *AOC3*), *OPR3*, *ACX1*; JA metabolism conversion related genes, including *ILL6, JAOs (JAO2, JAO3, JAO4), CYP94B1, CYP94B3, ST2A, JMT*; and JA signal transduction involved genes, like *JAZs* (*JAZ2*, *JAZ3*, *JAZ5*, *JAZ7*, *JAZ8*, *JAZ9*, *JAZ10*, *JAZ13*), were detected only or both in HW vs. WW and in HN vs. WW (Supplementary Figure 1 and Supplementary Tables 22, 23). These results implied that H_2_O_2_ pretreatment may decrease the levels of JA and its derivatives, but the JA signaling was still induced due to the decreased expression of many JAZs transcriptional repressors. Therefore, JA signaling plays an important role in the response of H_2_O_2_-pretreated Arabidopsis plants to subsequent salt stress. However, the action mechanisms of JA in plant salt stress tolerance remains largely elusive. Previous studies on different plants have given controversial conclusions (Qiu et al., 2014; Song et al., 2021), which means the roles of JA in plant salt stress tolerance are sophisticated. In this process, the combined action of JA with other plant hormones, such as ABA, ethylene, auxin, and salicylic acid, plus the regulation of hormonal homeostasis may jointly contribute to plant growth under salt stress (Raza et al., 2021; Zahedi et al., 2021; Zhao et al., 2021; Zhu et al., 2021).

Besides the abovementioned genes, many DEGs involved in the redox balance were induced separately in HW, HN, or in both conditions. These genes include *TT4*, *CRWN2*, *CRWN3*, *CRWN4*, *ACS6*, *SOS6*, *RBOHD*, *VTC2*, *FSD3*, *GPX7*, and *GSTU24*, which are able to scavenge ROS to maintain the redox balance; moreover, their up-regulated expression enhances oxidative stress tolerance in plants. Then, we examined the enzyme activity of the antioxidative enzymes responsible for the scavenging of ROS. SOD is an important protective enzyme in the enzymatic defense system, which can eliminate superoxide radicals in the cell through a dismutation reaction, generating H_2_O_2_ and O_2_ (Noctor and Foyer, 1998). Our experiments showed that SOD activity increased in the leaves of both HN and HW seedlings, suggesting that H_2_O_2_ pretreatment might enhance the superoxide radical scavenging ability of plants. It has been shown that salt tolerance is directly related to an increase in SOD activity (Hernandez et al., 2000). It is also noteworthy that the enhancement of SOD activity in HN plant leaves was accompanied by increases in GPX activity. These results indicate that under stressed conditions, the ROS scavenging mechanism was more effective in acclimated than in unacclimated plants. Thus, our results suggest that SOD and GPX may play central protective roles in the O_2_^–^ and H_2_O_2_ scavenging processes (Badawi et al., 2004; Gill and Tuteja, 2010), and that the active involvement of these enzymes is related, at least in part, to salt-induced oxidative stress tolerance in plants. In addition, we found that among the differentially up-regulated genes, the osmotic stress-responsive genes were mainly concentrated under HN conditions, while relatively few were associated with HW. *RD29A* and *RD29B* encode hydrophilic proteins which act as protective molecules in response to osmotic stress, and *P5CS1* encodes a key enzyme in proline biosynthesis to promote the accumulation of proline (Feng et al., 2016).

Besides keeping osmotic balance, ion homeostasis maintenance is also an important mechanism for salinity tolerance in plants. From our RNA-Seq data, we also found some genes, such as *NHX2*, *CAX3*, and *CIPK5* were upregulated under the condition of H_2_O_2_ pretreatment followed NaCl stress (Supplementary Table 23). *NHX2*, as a tonoplast-localized NHX isoform, contributes to both vacuolar pH and the uptake of K^+^ and Na^+^, therefore regulating intracellular ion homeostasis (Bassil et al., 2019). CAX3 is a vacuolar H^+^/Ca^2+^ antiporter, participating in vacuolar H^+^/Ca^2+^ transport during salt stress. Unlike *CAX1*, *CAX3* expression is strongly induced by salt stress (Cheng et al., 2003) and therefore has a specific role in response to salt stress (Zhao et al., 2008). Ion homeostasis, especially K^+^ homeostasis, is critical for metabolism, cell expansion, plant growth and plant stress acclimation. In this work, many up-regulated DEGs in HN are enriched in starch and sucrose metabolism, may partly benefiting from this K^+^ homeostasis. Through these pathways plants can improve source/sink of carbon, and therefore maintain their growth and development under salt stress.

The plant cell wall is mainly composed of cellulose, hemicellulose, and pectin. In addition, it contains enzymes and structural proteins. Plant cell walls are essential for the normal growth and development of plants, having many functions such as determining cell shape, maintaining normal water balance and expansion pressure, regulating the spread of macromolecules, and resisting a variety of abiotic stresses. Under high-salt stress, the cell wall is destroyed and the cells lose water and die which, in turn, affects the normal growth and development of plants. Therefore, cell wall integrity is critical for plants growth and stress response (Zhao et al., 2021). Similar to our previous research results, a low level of H_2_O_2_ pretreatment and subsequent high salinity, individually or jointly, induced the expression of cell wall remodeling genes. AtGH9B1, AtGH9B8, and AtGH9B13 are class B endoglucanases, and AtGH9C2 is a class C endoglucanase, all of which regulate the synthesis of cellulose. FUT4 is responsible for the fucosylation of AGPs in leaves, and its up-regulation has been shown to thicken plant cell walls and enhance plant salt tolerance (Tryfona et al., 2014).

Based on our work, we suppose that H_2_O_2_ pretreatment activates multiple stress-responsive signal pathways which are integrated into a signal network that initiates the expression of many genes encoding transcription factors and protein kinases, hence transitioning plants into a primed state for combatting future salt stress (Hossain et al., 2015).

## Conclusion

**FIGURE 7:**
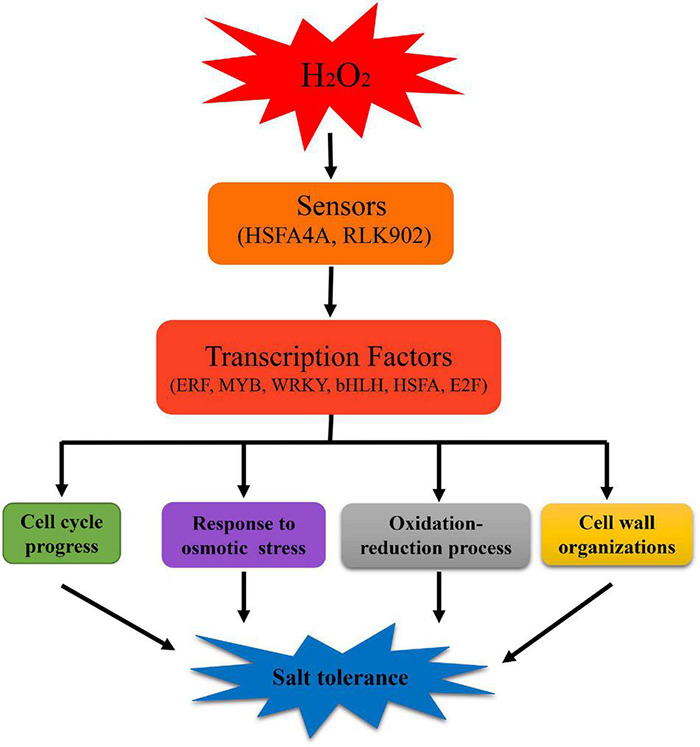
A working model of the mechanism underlying H_2_O_2_ pretreatment to improve salt tolerance in Arabidopsis. After H_2_O_2_ pretreatment, plants respond to salt stress via complex signal transduction pathways. We speculate that HSFA4A and RLK902 act as sensors in the H_2_O_2_ pretreatment process, transmitting the H_2_O_2_ signal to activate transcription factors, including those in WRKY, ERF, MYB, HSFA, bHLH, and E2F families. These transcription factors activate a series of genes with responses in cell cycle progress, osmotic stress, redox processes, and cell wall tissue, ultimately improving plant salt tolerance.

To summarize, under salt stress conditions, H_2_O_2_-pretreated plants displayed high-salt tolerance when compared to non-pretreated plants, as manifested in higher plant biomass, increased K^+^/Na^+^ ratio, declined MDA content and RMP decline, and elevated activities of antioxidative enzymes (including SOD, and GPX). These results were integrated with transcription data, and we propose a working model for H_2_O_2_ pretreatment-induced salt tolerance improvement of Arabidopsis plants. In brief, exogenous H_2_O_2_ may be perceived by certain sensors which transmit the H_2_O_2_ signal to transcription factors that, in turn, regulate the expression of downstream genes, thereby accelerating cell cycle progression and cell proliferation, enhancing osmotic stress tolerance, maintaining the redox balance, and remodeling the cell walls in plants under subsequent high-salt exposure. In accordance, pretreatment with H_2_O_2_ at an appropriate concentration can improve the growth and salt tolerance of Arabidopsis seedlings (Figure 7).

## Data Availability Statement

The datasets presented in this study can be found in online repositories. The names of the repository/repositories and accession number(s) can be found in the article/Supplementary Material.

## Author Contributions

QZ performed transcriptome analysis and wrote the manuscript. XD performed transcriptome analysis and made all heatmaps. HW and FW performed qRT-PCR experiments and modified the manuscript. DT performed plant material cultivation and RNA extraction. CJ and XZ measured physiological data. CM and HZ provided critical discussion. PL, YZ, and ZW proposed the idea of the manuscript, supervised the whole work, and wrote the final draft of the manuscript. All authors have read and agreed to the published version of the manuscript.

## Conflict of Interest

The authors declare that the research was conducted in the absence of any commercial or financial relationships that could be construed as a potential conflict of interest.

## Publisher’s Note

All claims expressed in this article are solely those of the authors and do not necessarily represent those of their affiliated organizations, or those of the publisher, the editors and the reviewers. Any product that may be evaluated in this article, or claim that may be made by its manufacturer, is not guaranteed or endorsed by the publisher.

## Footnotes

1
https://www.arabidopsis.org/

2
http://www.bioconductor.org/

## Abbreviations

APX: ascorbate peroxidase
AsA: ascorbate
CAT: catalase
EDTA: ethylene diamine tetraacetic acid
ET: ethylene
GPX: glutathione peroxidase
GR: glutathione reductase
GSH: glutathione
H_2_O_2_: hydrogen peroxide
HW: pretreated with H_2_O_2_ and not salt-stressed
HN: pretreated with H_2_O_2_ and salt-stressed
MDA: malonaldehyde
NBT: nitro blue tetrazolium chloride
POD: peroxidase
RMP: relative membrane permeability
ROS: reactive oxygen species
SOD: superoxide dismutase
WW: pretreated with water and not salt-stressed
WN: pretreated with water and salt-stressed.
