# Supplementary figures and images for: Transcriptomic Profiling Provides Molecular Insights Into Hydrogen Peroxide-Enhanced Arabidopsis Growth and Its Salt Tolerance
Source: Front Plant Sci. 2022 Apr 6;13:866063. doi: 10.3389/fpls.2022.866063 (PMC9019583; doi:10.3389/fpls.2022.866063)

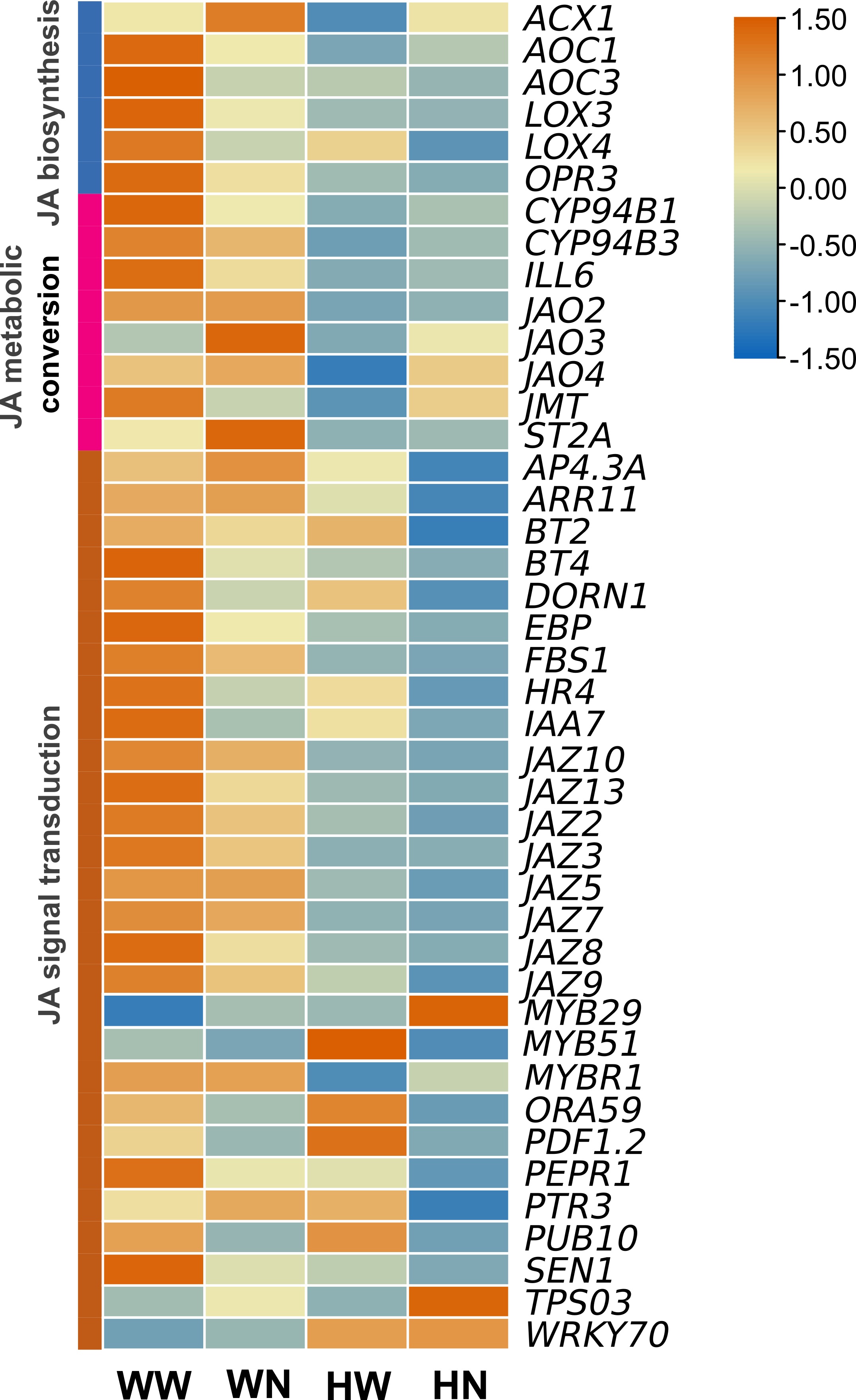

Supplement: Supplementary file 1 [file Image_1.JPEG]
