# Supplementary material for: Transcriptomic Profiling Provides Molecular Insights Into Hydrogen Peroxide-Enhanced Arabidopsis Growth and Its Salt Tolerance
Source: Front Plant Sci. 2022 Apr 6;13:866063. doi: 10.3389/fpls.2022.866063 (PMC9019583; doi:10.3389/fpls.2022.866063)
Supplement: Supplementary file 2 [file Data_Sheet_1.PDF]

Supplementary data

Figure S1 The expression patterns of some DEGs involved in JA biosynthesis, metabolism and signal transduction represented as a heatmap. WW, pretreated with water and not salt-stressed; WN, pretreated with water and salt-stressed; HW, pretreated with H_2O_2 and not salt-stressed; HN, pretreated with H_2O_2 and salt-stressed. Heat map diagram of the log2FC, the red and blue colors specify up-and down-regulated expressions.

Table S1 Primers used in verification of RNA-seq data by RT-qPCR.
Table S2 There are 1493 DEGs in HW vs. WW, in which 993 up-regulated and 500 down-regulated.
Table S3 There are 2467 DEGs in HN vs. WW, in which 1212 up-regulated and 1255 down-regulated.
Table S4 There are 1533 DEGs in WN vs. WW, in which 922 up-regulated and 604 down-regulated.
Table S5 There are 602 up-regulated DEGs only in HW vs. WW.
Table S6 There are 455 up-regulated DEGs only in HN vs. WW.
Table S7 There are 364 up-regulated DEGs only in WN vs. WW.
Table S8 There are 361 common up-regulated DEGs in HW vs. WW and HN vs. WW.
Table S9 There are 169 common up-regulated DEGs in HW vs. WW and WN vs. WW.
Table S10 There are 535 common up-regulated DEGs in HN vs. WW and WN vs. WW.
Table S11 There are 236 down-regulated DEGs only in HW vs. WW.
Table S12 There are 684 down-regulated DEGs only in HN vs. WW.
Table S13 There are 171 down-regulated DEGs only in WN vs. WW.
Table S14 There are 256 common down-regulated DEGs in HW vs. WW and HN vs. WW.
Table S15 There are 118 common down-regulated DEGs in HW vs. WW and WN vs. WW.
Table S16 There are 425 common down-regulated DEGs in HN vs. WW and WN vs. WW.
Table S17 There are1766 DEGs in HN vs. WW, in which 780 up-regulated and 986 down-regulated. We deduct DEGs from HN vs. WW that are consistent with the abundance of WN vs. WW expression, that is $0.67 < \log 2FC$ (HN vs. WW) / $\log 2FC$ (WN vs. WW) < 1.5. .
Table S18 Functional classification of up-regulated DEGs in HW vs. WW.
Table S19 Functional classification of down-regulated DEGs in HW vs. WW.
Table S20 Functional classification of up-regulated DEGs in HN vs. WW.
Table S21 Functional classification of down-regulated DEGs in HN vs. WW.
Table S22 DEGs involved in JA biosynthesis, metabolism and signal transduction, and ion transport and homeostasis in HW vs. WW.
Table S23 DEGs involved in JA biosynthesis, metabolism and signal transduction, and ion transport and homeostasis in HN vs. WW.
